# MicroRNA-455-3p regulates proliferation and osteoclast differentiation of RAW264.7 cells by targeting PTEN

**DOI:** 10.1186/s12891-022-05266-0

**Published:** 2022-04-09

**Authors:** Xiaolu Zhang, Liangming Wang, Nianlai Huang, Yiqiang Zheng, Liquan Cai, Qingfeng Ke, Shiqiang Wu

**Affiliations:** 1 The Second Affiliated Hospital of Fujian Medical University, Quanzhou, 362000 China; 2 The Second Clinical Medical College, Fujian Medical University, Fuzhou, 350000 China

**Keywords:** miR-455-3p, PTEN, RAW264.7, Cell proliferation, Osteoclast differentiation

## Abstract

**Background:**

Macrophages are one of the important cells in immune system. In this article, we aim to explore the regulatory role of miR-455-3p on proliferation and osteoblast differentiation of RAW264.7 cells.

**Methods:**

Expression levels of genes and proteins in cells were tested via qRT-PCR and western blot. The targeted correlation between miR-455-3p and PTEN was identified by luciferase analysis. MTT assay and flow cytometry were applied to detect the proliferation and apoptosis of cells. Osteoclastogenesis was completed by stimulating RAW 264.7 cells with RANKL. Tartrate-resistant acid phosphatase (TRAP) activity in different groups of cells were assessed.

**Results:**

Firstly, we determined that up-regulation of miR-455-3p promoted the proliferation and inhibited apoptosis of RAW 264.7 cells. MiR-455-3p deficiency played opposite effect in RAW 264.7 cells. Additionally, osteoclastogenesis-related factors (TRAP, CTSK and NFATc1) expression levels were remarkably up-regulated in miR-455-3p-mimic group of RAW264.7 cells treated with RANKL, but decreased in inhibitor group. Luciferase assay proved that miR-455-3p targeted PTEN. We took a further step and found overexpression of PTEN significantly inhibited the increased proliferation and osteoblast differentiation of RAW264.7 cells induced by miR-455-3p.

**Conclusions:**

Our findings supported basic to explore the molecular mechanism of proliferation and osteoblast differentiation of RAW264.7 cells.

**Supplementary Information:**

The online version contains supplementary material available at 10.1186/s12891-022-05266-0.

## Background

In immune system, macrophages belonging to the important cells. Proliferation and differentiation ability of macrophage plays important role in the innate immune system and a prerequisite for immune and inflammatory responses [1]. In hematopoietic system, macrophages are one of the most plastic cells. They exist in a large number of bodies and tissues and have many functions. Macrophages play multiple roles in growth, homeostasis, wound recovery, and immune response to exogenous substances [2]. At the same time, deregulated proliferation and differentiation of macrophages has been implicated in some diseases, such as osteoporosis, cancer, inflammatory and neurodegenerative diseases [2–4]. In the study of macrophage function, RAW 264.7 is a model cell used by a large number of researchers in the evaluation of immune response and macrophage differentiation [5]. Liyanage et al. used RAW 264.7 cells to research the biological function of porcine gastric mucin in stress resistance and immune regulation [6]. Kim and his partner used RAW 264.7 cells treated with RANKL to explore the function of zanthoxylum piperitum in the bone loss in osteoporosis [7]. These studies suggest that the molecular mechanism of abnormal proliferation and differentiation of RAW 264.7 cells are of great significance.

As small non-coding RNAs, microRNAs perform vital function in various biological processes [8, 9]. MiRNAs contribute to a variety of biological functions of macrophages including RAW 264.7 cells [10–12]. MiR-146a has been shown that in RAW264.7 cell line macrophages are polarized by inhibiting the Notch1 pathway [13]. For the differentiation of RAW264.7, Li and his partners found the formation of osteoclasts could be affected by the abnormal expression of miR-133a [14]. Wang et al. found the differentiation of RAW264.7 cell line into osteoclasts could be inhibited by up-regulation of miR-218 or miR-618 [15]. In recent years, a large number of scientists have begun to pay attention to the function and mechanism of miRNA on RAW264.7 cell. The abnormal expression of miR-455-3p plays an important role in many diseases. In a recent study, the expression of miR-455-3p has been reported was abnormal in the process of osteoclast and osteoblast differentiation. Notably, Zhang and his partners found miR-455-3p/Nrf2/ARE signaling axis is important in inhibiting the oxidative stress of osteoclasts [16]. For RAW264.7 cells, miR-455-3p expression level has also been proved higher in RANKL-induced RAW264.7 cells compared with normal cells [17]. However, the specific effect of the abnormal expression of Mir-455-3p on the RAW264.7 cell line is not clear.

In the present research, miR-455-3p has been found up-regulation in RANKL induced RAW264.7 cells. Up-regulation of miR-455-3p promoted the growth and osteoclast activity of RAW264.7 cell line and down-regulated expression of miR-455-3p increased the apoptosis rate of RAW264.7 cell line. Moreover, our research proved miR-455-3p might regulate the transcription of PTEN to influence the proliferation and cell cycle of RAW264.7 cells and osteoclast formation.

## Methods

### The culture conditions of cell

RAW264.7 cell line were acquired from the American Type Culture Collection (ATCC, Rockville, USA). The culture condition of cells was 10% fetal bovine serum (Gibco), 100 U/ml penicillin and 100 mg/ml streptomycin (gibco) added into Dulbecco’s modified Eagle medium (DMEM; gibco, grand island, NY, USA). For osteoclastogenesis, RANKL (50 ng/ml) were used to treated RAW 264.7 cells for 7 d in control group. For treatment group, M-CSF (50 ng/ml) and RANKL (50 ng/ml) were used to treated RAW 264.7 cells for 7 d. The medium was changed three times (less than 48 h). Cells were cultured at 37 °C, 5% CO_2_ in a humidified atmosphere.

### The method of cell transfection

Over-expression of PTEN gene (oe-PTEN) and the negative controls were procured ordered from GenePharma (Shanghai, China). MiR-455-3p mimic-F: 5′-GCAGUCCA UGGGCAUAUACAC-3′, miR-455-3p mimic-R: 5′-GUAUAUGCCCAUGGACUGC UU-3′, miR-455-3p inhibitor-F: 5′-GUGUAUAUGCCCAUGGACUGC-3′, miR mimic -NC-F: 5′-UUCUCCGAACGUGUCACGUTT-3′, miR mimic-NC-R: 5′-ACGUGACACGUUCGGAGAATT-3′ and miR inhibitor-NC 5′-CAGUACUUUUGUGUAGUACAA-3′ were synthesized by Genepharma (Shanghai, China). These genes and negative controls (50 nM) were transfected into cells, respectively. In the rescue experiments, over-expression (oe) of the PTEN gene (oe-PTEN) and oe-NC were respectively added into mimic or NC of miR-455-3p to transfected into cells. Lipofectamine2000 (Thermo Fisher Scientific, Waltham, USA) was applied for cell transfection, following the manufacturer’s instructions.

### The method of RNA expression detection

For RNA extracted and cDNA obtained, we used TRIzol and All-in-One First-Strand cDNA Synthesis kit (Genecopoeia, Rockville, MD, USA) according to instruction manual. The expression of miRNA levels was detected by miScript SYBR Green PCR Kit (Qiagen, Germany). SYBR premix Ex Taq TM II (Takara Bio Inc., Japan) was used to detect mRNA expression levels. qPCR primers used: miR-455-3p-F: 5′-TAAGACGTCCATGGGCAT-3′, miR-455-3p-R: 5′-GTGCAGGGTCCGAGGT-3′, PTEN-F: 5′-ACCAGTGGCACTGTTGTTTCAC-3′, PTEN-R: 5′-TTCCTCTGGTCCTGGTATGAAG-3′, GAPDH-F: 5′-GGAGCGAGATCCCTCCAAAAT-3′, GAPDH-R: 5′-GGCTGTTGTCATACTTCTCATGG-3′, U6-F: 5′-CTCGCTTCGGCAGCACA, U6-R: 5′-AACGCTTCACGAATTTGCGT-3′. U6 and GAPDH were used as internal parameters of miRNA and mRNA, respectively. The method of 2^-ΔΔCt^ was used to analyzed the results of expression.

### Analytical method of western blot

RIPA buffer, protease inhibitor and phosphatase inhibitor were used to treated cells. 2D quantitative assay kit (GE) was used for measurement of protein concentration. A 25 μg protein sample was separated by SODIUM dodecyl sulfate polyacrylamide gel electrophoresis (SDS-PAGE) for 45 min and then transferred to a cellulose nitrate membrane. The membranes were blocked with 5% skim milk for 2 h. Under a condition of 4 °C, the antibodies of PTEN (ab267787, 1:1000; abcam), TRAP (ab52750, 1:5000; abcam), CTSK (ab207086, 1:1000; abcam), NFATc1 (MA3–024, 1:20, invitrogen), p-AKT (4060, 1: 1000, CST), CyclinD1 (ab16663, 1:200, abcam) and GAPDH (LS-B4075; 1:5000; LifeSpan Biosciences) were treated overnight with membranes. Afterwards, TBST was used to treated the membranes three times and then hybridized with IgG (ab205718, 1:2000; abcam).

### MTT assay

MTT assay was carried out to detect cell growth. Three repeated wells were set for each treatment. MTT cell proliferation and cytotoxicity assay Kit (Beyotime, China) was used to detected the cell growth. At 0,1, 2, 3 and 4 d, 10 μl of MTT (5 mg/ml) was added into the cell line, and then incubated at 37 °C for 4 h, after which 100 μl solubilizing buffer was used to incubated the cell line overnight at 37 °C. At last, Spectrophotometer at 490 nm was used to analyzed the results (Molecular Devices, USA).

### Apoptosis and cycle experiments

Flow cytometry was applied for assessing cell apoptosis. After transfection, cells (3 × 10^5^ cells/well) were placed in a 6-well plate. 5 μl Annexin V-FITC/PI (20%; Invitrogen, USA.) was used to stained the cells. Finally, apoptotic cell rate was determined by flow cytometry (BD, Biosciences). Each experiment was repeated in triplicate.

RAW264.7 cells were directly collected into a 10 mL centrifuge tube, and the concentration of cells was 3 × 10^6^ cells/mL per sample. The cells were dyed with propyl iodide (PI), and the content of DNA was measured with a flow cytometry (BD, Biosciences). ModFit software was used to analyzed the results of cell cycle.

### Prediction of downstream Target Genes of miR-455-3p and Double luciferase reporting experiment

The target genes of miR-455-3p were predicted using miRTarBase (https://mirtarbase.cuhk.edu.cn/) and miRDB (http://mirdb.org/). For a relatively robust selection of target genes, the overlapped target genes from the above two databases were detected by Venn diagram analysis. The binding site of miR-455-3p in 3′-untranslated regions of PTEN was predicted using TargetScan (http://www.targetscan.org/vert_72/).

The PTEN 3′-UTR was amplified by PCR using the following primers: F 5′-GATCGCTCGAGTTTCAATCATAATACCTGC-3′, R 5′-GCGGCCAGCGGCCGC TTCTGCCAAATACTACAGTTA-3′. The seed sequences were mutated using the following primers: F 5′-ACTGTGTTTGTGAGCCCCTCCTTCCCACCGGAAGTC CAGCTTCA-3′, R 5′-TGAAGCTGGACTTCCGGTGGGAAGGAGGGGCTCACA AACACAGT-3′. Luciferase reporter vectors were constructed with 3′ UTR regions of WT and MUT PTEN. PTEN-wt/mut binding with NC/miR-455-3p mimic was co-transfected in RAW264.7 cell line. PRL-TK vector (TaKaRa, Dalian, China) as an internal reference. Lastly, the activity of luciferase was determined by luciferase assay reagent.

### The method of TRAP staining

RAW264.7 cells (1 × 10^4^ cells/well) were seeded into sterile 24-well culture plates, cells were fixed and stained using the Acid Phosphatase Leukocyte kit (Sigma-Aldrich St Louis, USA), according to the manufacturer’s instructions. Distilled water was used to washed cells and staining reagents were removed. The definition of osteoclasts was multinucleated cells including more than 3 nuclei. Photomicrographs were taken with Ziess AxioCam digital microscope (Zeiss, Oberkochen, Germany).

### Statistical analysis

Graphpad 6.0 statistical software was used to analyzed the data and data were represented as mean ± SD. T-test was used to analyze the data between 2 groups. For comparisons among three or more groups, one-way ANOVA with Tukey post hoc test was used. *P* value < 0.05 represented statistically significant. All experiments were repeated in triplicate.

## Results

### Cell proliferation, apoptosis and osteoclast formation of RAW264.7 cells were regulating with abnormal expression of miR-455-3p

**Fig. 1:**
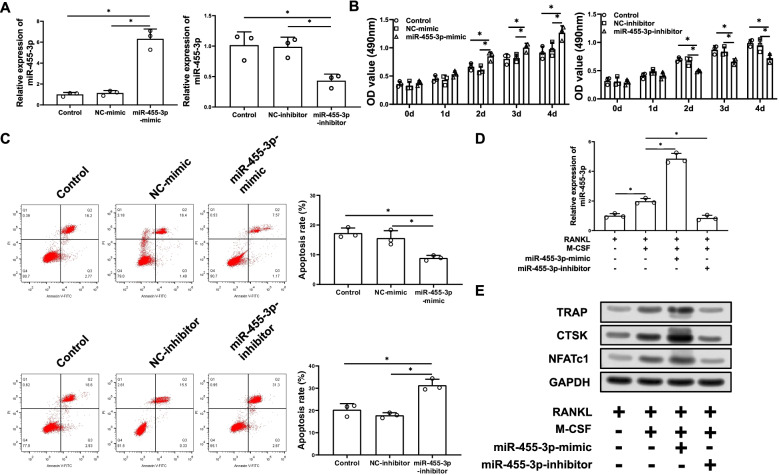
Cell proliferation and osteoclast formation of RAW264.7 cells were regulated with miR-455-3p. **A** qRT-PCR showed the relative expression of miR-455-3p in RAW264.7 cells treated with miR-455-3p-mimic or miR-455-3p-inhibitor; **B** MTT assay for detection of cell proliferation; **C** Flow cytometry for detection of cell apoptosis; **D** The relative mRNA level of miR-455-3p in different groups of RAW264.7 cell line was measured; **E** The protein levels of osteoclast formation-related factors (TRAP, NFATc1 and CTSK) was measured; * represented *P* < 0.05

Mimic and inhibitor of miR-455-3p and their control were transfected into cell line, respectively. Transfection efficiency was detected by qRT-PCR. Compared with control (Control: 1.00 ± 0.15) and NC-mimic groups (NC-mimic: 1.14 ± 0.19), miR-455-3p was successfully increased in the cells transfected with miR-455-3p-mimic (*p* = 0.008 compared with control group; *p* = 0.008 compared with NC-mimic group). Compared with control (Control: 1.00 ± 0.18) and NC-inhibitor groups (NC-inhibitor: 0.99 ± 0.13), miR-455-3p was successfully decreased in the cells transfected with miR-455-3p inhibitor (*p* = 0.027 compared with control group; *p* = 0.010 compared with NC-inhibitor group) (Fig. 1A). Then cell biological behaviors were assayed via cell experiments. MTT assay suggested that compared with control (Control 0 d: 0.36 ± 0.04, Control 1 d: 0.45 ± 0.05, Control 2 d: 0.66 ± 0.05, Control 3 d: 0.80 ± 0.07, Control 4 d: 0.91 ± 0.09) and NC-mimic groups (NC-mimic 0 d: 0.33 ± 0.06, NC-mimic 1 d: 0.43 ± 0.07, NC-mimic 2 d: 0.60 ± 0.06, NC-mimic 3 d: 0.82 ± 0.06, NC-mimic 4 d: 0.98 ± 0.10), up-regulation of miR-455-3p resulted in the increase of cell proliferation ability (*p* = 0.034 compared with control 2 d; *p* = 0.044 compared with control 3 d; *p* = 0.022 compared with control 4 d; *p* = 0.016 compared with NC-mimic 2 d; *p* = 0.036 compared with NC-mimic 3 d; *p* = 0.045 compared with NC-mimic 4 d). Compared with control (Control 0 d: 0.32 ± 0.05, Control 1 d: 0.41 ± 0.04, Control 2 d: 0.69 ± 0.03, Control 3 d: 0.87 ± 0.06, Control 4 d: 0.99 ± 0.05) and NC-inhibitor groups (NC-inhibitor 0 d: 0.31 ± 0.07, NC-inhibitor 1 d: 0.48 ± 0.04, NC-inhibitor 2 d: 0.67 ± 0.06, NC-inhibitor 3 d: 0.84 ± 0.07, NC-inhibitor 4 d: 0.95 ± 0.09), down-regulation of miR-455-3p resulted in the decrease of cell proliferation ability (*p* = 0.004 compared with control 2 d; *p* = 0.019 compared with control 3 d; *p* = 0.011 compared with control 4 d; *p* = 0.045 compared with NC-inhibitor 2 d; *p* = 0.041 compared with NC-inhibitor 3 d; *p* = 0.050 compared with NC-inhibitor 4 d) (Fig. 1B). Meanwhile, in Fig. 1C presented that cell apoptotic rate (Control: 17.28 ± 1.40, NC-mimic: 15.60 ± 2.04) in miR-455-3p-mimic treated cells was greatly decreased (*p* = 0.005 compared with control group; *p* = 0.033 compared with NC-mimic group) and cell apoptotic rate (Control: 20.35 ± 2.22, NC-inhibitor: 17.83 ± 0.96) in miR-455-3p-inhibitor (*p* = 0.007 compared with control group; *p* = 0.005 compared with NC-inhibitor group) treated cells was greatly increased. Taken together, these results indicate that the abnormal expression of miR-455-3p significantly affects the proliferation and apoptosis of RAW264.7 cell line.

To define the function of miR-455-3p during osteoclast formation, RAW264.7 cells were treated with RANKL/M-CSF. MiR-455-3p expression (RANKL induced RAW264.7 group: 1.00 ± 0.12) is upregulated in the process of RANKL/M-CSF induced RAW264.7 cells (*p* = 0.003 compared with RANKL induced RAW264.7 group) (Fig. 1D). For overexpression and inhibit efficiency of miR-455-3p, the expression of miR-455-3p in RANKL/M-CSF induced RAW264.7 cells (RANKL/M-CSF induced RAW264.7 group: 1.98 ± 0.16) was promoted in the group of miR-455-3p-mimic (*p* = 0.001 compared with RANKL/M-CSF induced RAW264.7 group) and reduced in the group of miR-455-3p-inhibitor (*p* = 0.002 compared with RANKL/M-CSF induced RAW264.7 group) (Fig. 1D). Meanwhile, changes in the protein levels of several osteoclast related factors (TRAP, NFATc1 and CTSK) were measured. As shown in Fig. 1E, the protein levels of osteoclast related factors were significantly upregulated in RANKL/M-CSF-induced RAW264.7 cells compared with control cells (Fig. 1E). In addition, osteoclast formation-related factors protein levels were significantly upregulated in miR-455-3p overexpression group, but reduced in inhibitors group (Fig. 1E). Collectively, it was fully indicated that miR-455-3p might positively regulates osteoclastogenesis.

### MiR-455-3p directly targets PTEN

**Fig. 2:**
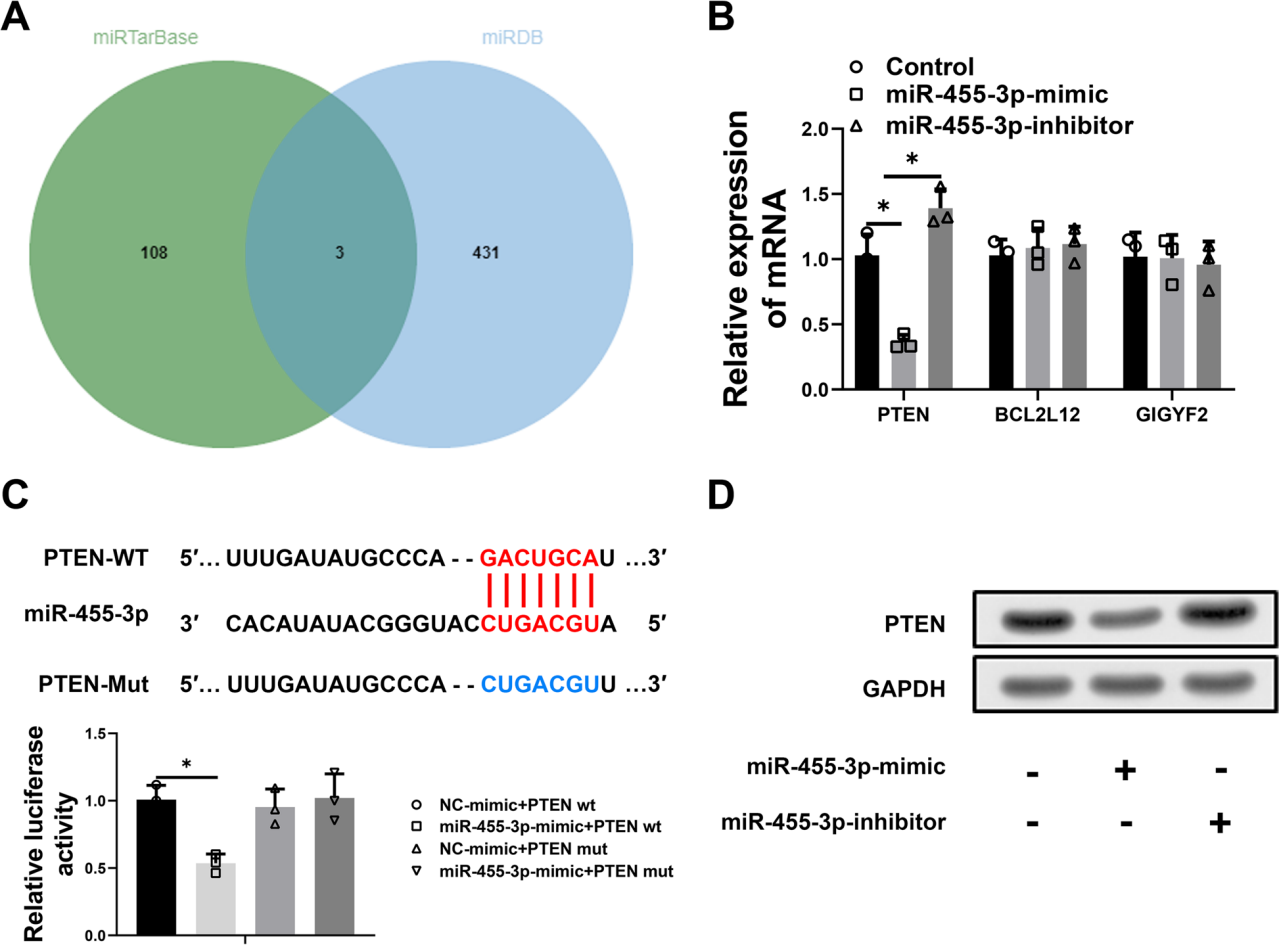
The binding relationship between miR-455-3p and PTEN. **A** Venn diagram of predicted mRNA binding with miR-455-3p with miRTarBase and miRDB; **B** The expression levels of the 3 genes were measured; **C** The direct targeted relationship between miR-455-3p and PTEN was assessed; **D** The protein level of PTEN in different groups of RAW264.7 cells was measured; * represented *P* < 0.05

Then, we used online sites to predict that miR-455a-3p might target binding mRNAs, including miRTarBase and miRDB (Supplement Table 1). We found miR-455-3p might target 3 mRNAs (PTEN, BCL2L12 and GIGYF2) (Fig. 2A). To find the mRNA most likely to bind miR-455-3p, the change of expression levels of these mRNAs in miR-455-3p-mimic (PTEN in miR-455-3p-mimic: 0.36 ± 0.05, BCL2L12 in miR-455-3p-mimic: 1.09 ± 0.12, GIGYF2 in miR-455-3p-mimic: 1.01 ± 0.15), miR-455-3p-inhibitor (PTEN in miR-455-3p-inhibitor: 1.39 ± 0.12, BCL2L12 in miR-455-3p-inhibitor: 1.12 ± 0.11, GIGYF2 in miR-455-3p-inhibitor: 0.96 ± 0.15) or control groups (PTEN in control: 1.00 ± 0.13, BCL2L12 in control: 1.00 ± 0.10, GIGYF2 in control: 1.00 ± 0.15) were measured. The results showed only the expression of PTEN was changed (*p* = 0.011 miR-455-3p-mimic compared with control group; *p* = 0.045 miR-455-3p-inhibitor compared with control group) (Fig. 2B). Then, the binding sites of PTEN 3′-UTR and miR-455-3p was measured with TargetScan and binding relationship between miR-455-3p and PTEN was analyzed by luciferase assay (NC-mimc + PTEN wt: 1.00 ± 0.09, miR-455-3p-mimc + PTEN wt: 0.54 ± 0.06, NC-mimc + PTEN mut: 0.95 ± 0.11, miR-455-3p-mimc + PTEN mut: 1.02 ± 0.15, *p* = 0.005 NC-mimc + PTEN wt compared with miR-455-3p-mimc + PTEN wt) (Fig. 2C, Supplement Fig. 1). The protein level of PTEN affected by miR-455-3p was also measured by western blot. As shown in Fig. 2D, protein level was remarkably up-regulated in miR-455-3p-inhibitor group, but decreased in mimic group. All these findings suggest that miR-455-3p is a negative regulator of PTEN.

### MiR-455-3p/PTEN axis regulates biological function of RAW264.7 cell line

**Fig. 3:**
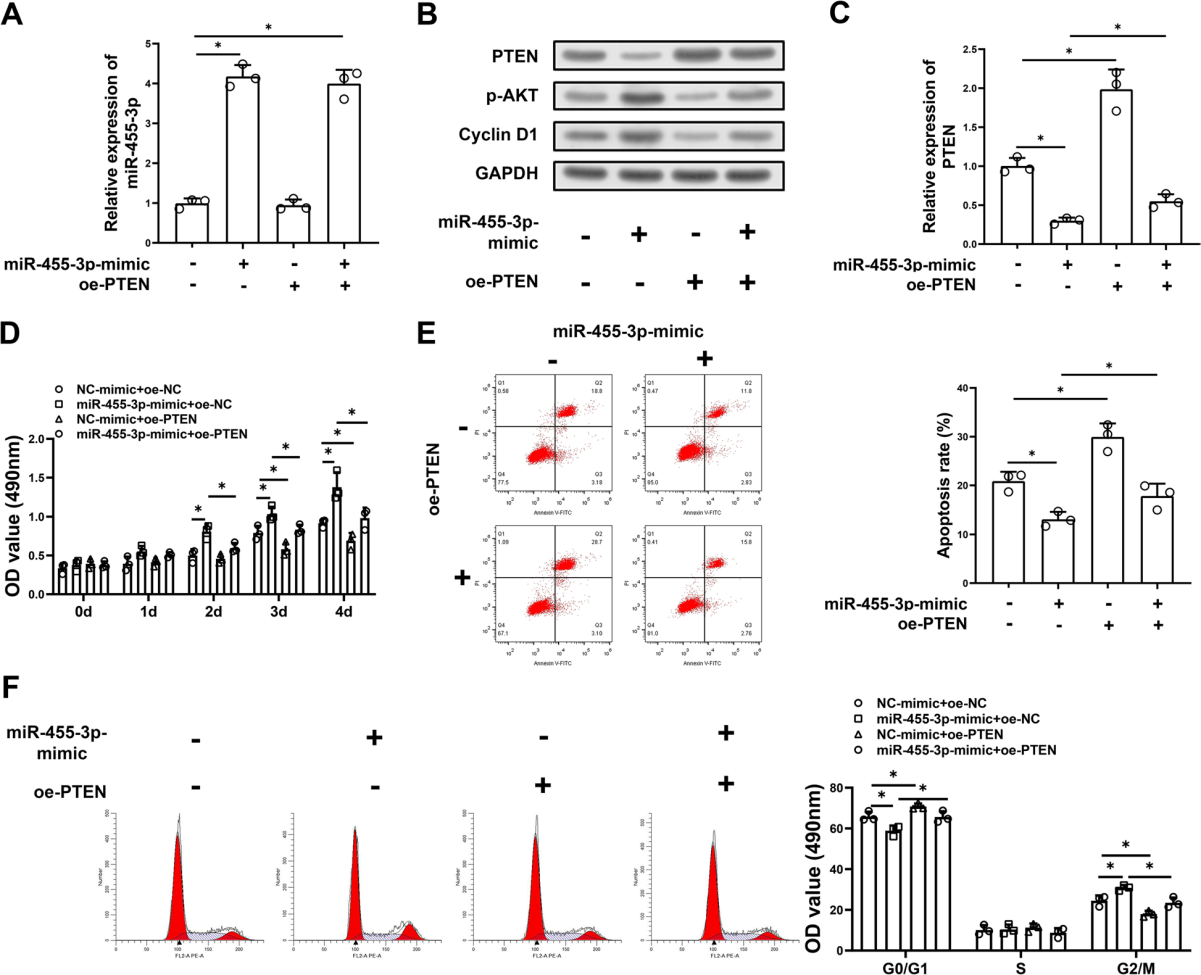
MiR-455-3p/PTEN axis regulates biological function of RAW264.7 cell line. **A** qRT-PCR showed the relative mRNA level of miR-455-3p in different groups of cells; **B** The protein level of PTEN, p-AKT and Cyclin D1 in different groups of RAW264.7 cells were assessed; **C** The relative mRNA expression of PTEN in different groups of RAW264.7 cells were assessed; **D** MTT assay for detection of cell proliferation; **E**-**F **Flow cytometry for detection of cell apoptosis and cell cycle; * represented *P* < 0.05, oe represented over-expression

To demonstrate that the miR-455-3p /PTEN signaling axis can regulate the biological function of RAW264.7 cells, NC-mimic + oe-NC, miR-455-3p-mimic + oe-NC, NC-mimic + oe-PTEN and miR-455-3p-mimic + oe-PTEN cell lines were constructed. MiR-455-3p (NC-mimic + oe-NC: 1.00 ± 0.10, miR-455-3p-mimic + oe-NC: 4.18 ± 0.23, NC-mimic + oe-PTEN: 0.95 ± 0.12, miR-455-3p-mimic + oe-PTEN: 4.00 ± 0.28) and PTEN (NC-mimic + oe-NC: 1.00 ± 0.08, miR-455-3p-mimic + oe-NC: 0.30 ± 0.03, NC-mimic + oe-PTEN: 1.98 ± 0.21, miR-455-3p-mimic + oe-PTEN: 0.55 ± 0.07) expression in different groups were assessed by qRT-PCR. PTEN protein level in different groups was measured by western blot. The results further illustrated that miR-455-3p (*p* < 0.001 miR-455-3p-mimic + oe-NC compared with NC-mimic + oe-NC; *p* = 0.002 miR-455-3p-mimic + oe-PTEN compared with NC-mimic + oe-NC) could inhibit the expression of PTEN (*p* = 0.003 miR-455-3p-mimic + oe-NC compared with NC-mimic + oe-NC; *p* = 0.012 NC-mimic + oe-PTEN compared with NC-mimic + oe-NC; *p* = 0.025 miR-455-3p-mimic + oe-PTEN compared with miR-455-3p-mimic + oe-NC) (Fig. 3A-C). MTT assay (NC-mimic + oe-NC 0 d: 0.34 ± 0.05, NC-mimic + oe-NC 1 d: 0.39 ± 0.08, NC-mimic + oe-NC 2 d: 0.50 ± 0.07, NC-mimic + oe-NC 3 d: 0.79 ± 0.08, NC-mimic + oe-NC 4 d: 0.92 ± 0.04) proved that up-regulation of miR-455-3p markedly enhanced growth of RAW264.7 cells (*p* = 0.012 miR-455-3p-mimic + oe-NC compared with NC-mimic + oe-NC 2 d; *p* = 0.034 miR-455-3p-mimic + oe-NC compared with NC-mimic + oe-NC 3 d; *p* = 0.044 miR-455-3p-mimic + oe-NC compared with NC-mimic + oe-NC 4 d), while overexpressing PTEN significantly attenuated the promoting effect of miR-455-3p on RAW264.7 cell line (*p* = 0.034 miR-455-3p-mimic + oe-PTEN compared with miR-455-3p-mimic + oe-NC 2 d; *p* = 0.046 miR-455-3p-mimic + oe-PTEN compared with miR-455-3p-mimic + oe-NC 3 d; *p* = 0.049 miR-455-3p-mimic + oe-PTEN compared with miR-455-3p-mimic + oe-NC 4 d) (Fig. 3D). The apoptosis and cycle of RAW264.7 cell was also test by flow cytometry. The results plotted in Fig. 3E presented that cell apoptotic rate in miR-455-3p overexpression cells (miR-455-3p-mimic + oe-NC: 13.10 ± 1.25) was greatly decreased compared with control group (NC-mimic + oe-NC: 20.87 ± 1.57) and apoptotic ability in miR-455-3p-mimic+oe-PTEN group was greatly increased compared with miR-455-3p-mimic+oe-NC group (*p* = 0.006 miR-455-3p-mimic + oe-NC compared with NC-mimic + oe-NC; *p* = 0.013 NC-mimic + oe-PTEN compared with NC-mimic + oe-NC; *p* = 0.042 miR-455-3p-mimic + oe-PTEN compared with miR-455-3p-mimic + oe-NC). For cell cycle, miR-455-3p-mimic could block cell cycle in G2/M phase, while overexpressing PTEN significantly attenuated the effect of miR-455-3p on RAW264.7 cells (G0/G1: *p* = 0.026 miR-455-3p-mimic + oe-NC compared with NC-mimic + oe-NC, *p* = 0.049 NC-mimic + oe-PTEN compared with NC-mimic + oe-NC, *p* = 0.038 miR-455-3p-mimic + oe-PTEN compared with miR-455-3p-mimic + oe-NC; G2/M: *p* = 0.033 miR-455-3p-mimic + oe-NC compared with NC-mimic + oe-NC, *p* = 0.029 NC-mimic + oe-PTEN compared with NC-mimic + oe-NC, *p* = 0.021 miR-455-3p-mimic + oe-PTEN compared with miR-455-3p-mimic + oe-NC) (Fig. 3F). In addition, the protein level of p-AKT and Cyclin D1 which could be regulated by miR-455-3p and PTEN in other cells was also measured by western blot. The results showed that up-regulation of miR-455-3p markedly promoted the expression of p-AKT and Cyclin D1 and overexpression of PTEN significantly attenuated the effect of miR-455-3p on the expression of p-AKT and Cyclin D1 (Fig. 3B). Taken together, these results suggest that miR-455-3p regulates the biological function of RAW264.7 cells by down-regulating the expression of PTEN.

### miR-455-3p regulates osteoclastogenesis through PTEN

**Fig. 4:**
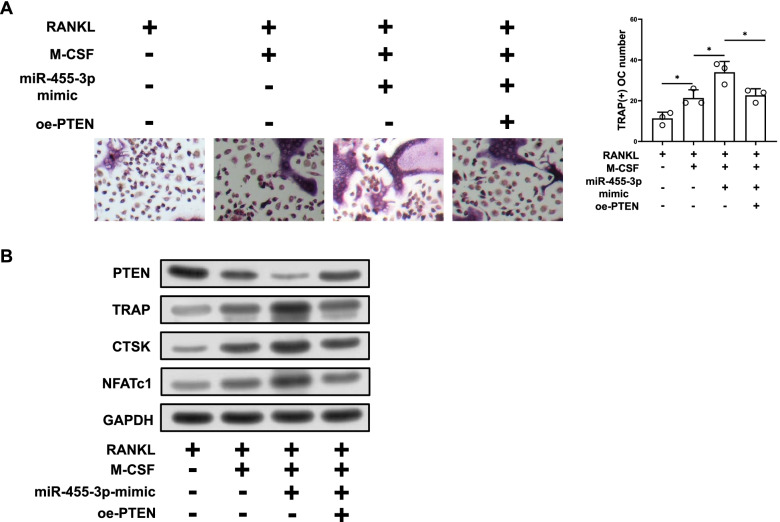
miR-455-3p regulates osteoclastogenesis through PTEN. **A** The TRAP positive rate in different group of RAW264.7 cells (original magnification × 200); **B** The protein levels of osteoclast formation -related factors (TRAP, NFATc1 and CTSK) in different groups of RAW264.7 cells was assessed; * represented *P* < 0.05, oe represented over-expression

Since miR-455-3p has been proved target PTEN, RAW264.7 cells treated with RANKL/M-CSF were used to contract miR-455-3p overexpression cells and miR-455-3p/PTEN overexpression cells. The TRAP results in RANKL/M-CSF induced cell line (RANKL induced cell group: 11.33 ± 2.49, RANKL/M-CSF induced cell group: 21.33 ± 3.30; *p* = 0.030 RANKL/M-CSF induced cell group compared with RANKL induced cell group) was significantly increased as compared with RANKL induced RAW264.7 cells (Fig. 4A). The group of miR-455-3p mimic showed higher TRAP-positive cells compared with the group of RANKL/M-CSF induced RAW264.7 cells (miR-455-3p mimic group: 34.00 ± 4.32; *p* = 0.033 miR-455-3p mimic group compared with RANKL/M-CSF induced cell group), while overexpressing PTEN significantly attenuated the promoting effect of miR-455-3p on osteoclast differentiation (miR-455-3p mimic + oe-PTEN group: 22.67 ± 2.62; *p* = 0.044 miR-455-3p mimic group compared with RANKL/M-CSF induced cell group) (Fig. 4A). The changes of protein levels of several osteoclast formation related factors were also measured, as shown in Fig. 4B, in the miR-455-3p overexpression group, osteoclast formation related factor protein levels were significantly upregulated, but overexpressing PTEN significantly inhibited the promoting function of miR-455-3p on protein level of osteoclastogenesis-related factors. Our experiments indicated that up-regulation of miR-455-3p promoted differentiation of RAW264.7 cells into osteoclast by targeting PTEN.

## Discussion

Being related to the processes of multiple cancers, the function of miR-455-3p in a variety of cancers has been studied [18, 19]. However, the function of miR-455-3p of differentiation and proliferation in RAW264.7 cell line has not been investigated so far. In the present researches, up-regulation of miR-455-3p in RAW264.7 cells significantly increased the cell proliferation and osteoclast formation, meanwhile down-regulation of miR-455-3p reduced proliferation and differentiation of RAW264.7 cells into osteoclasts. In addition, overexpression of PTEN can effectively reverse the promotion effect of miR-455-3p on RAW264.7 cell proliferation, cell cycle and osteoclast differentiation. Therefore, the present researches proved that miR-455-3p regulated the expression of PTEN and played a positive function on the proliferation of RAW264.7 cells and osteoclast differentiation.

The relationship between miRNAs and osteoclastogenesis was first demonstrated by Sugatani and his partners. They found miR-223 could inhibit osteoclastogenesis of RAW264,7 cells [20]. After that, some miRNAs have been found aberrant expressed and showed important roles in the osteoclast formation. Sun and his partners performed microarray detection in M-CSF/RANKL induced RAW 264.7 cells or not induced cells. The researches proved in the differentiation process of RAW 264.7 osteoclast precursor cells into osteoclasts, 44 microRNAs were differentially expressed [9]. Mao et al. found miR-346-3p promoted the regulation of osteoclast formation by inhibiting TRAF3 gene. They thought that miR-346-3p may be a novel therapeutic target for the pathogenesis of bone loss [21]. Similarly, a previous study has provided evidence that miR-506-3p could act as a molecular intervention for osteoclast formation mediated by RANKL/NFATc1 [22]. In this study, our results demonstrated that miR-455-3p could promoted the growth and inhibited the apoptosis of RAW 264.7 cell line. In addition, miR-455-3p was remarkably up-regulation in M-CSF/RANKL induced RAW 264.7 cells. Meanwhile, the expression levels of osteoclastogenesis-related factors were up-regulation in cells that were added in miR-455-3p-mimic and were reduced by anti-miR-455-3p treatment. Our present results suggested miR-455-3p paly a promoting effect on proliferation of RAW264.7 cells and osteoclastogenesis.

Many reports have shown that miRNAs bind to 3 ‘-UTR of target mRNAs to mediate negative regulation of gene expression. Our researches demonstrated PTEN could be regulated by miRNA-455-3p, and could be the reason of the effect of miR-455-3p on promoting proliferation of RAW264.7 cells and osteoclast differentiation. PTEN could regulate PI3K/AKT signaling pathway which has also been considered as a tumor suppressor [23]. PTEN has been proved to regulate cell proliferation, differentiation and cell cycle of several cancers and orthopedic diseases. In gallbladder cancer, PTEN has been proved to regulate AKT axis to suppress proteasome activity and bortezomib sensitivity [24]. In breast cancer, the absence of PTEN may predict more aggressive behavior and worse outcomes in breast cancer patients [25]. Except for cancers, PTEN also function as a regulatory factor in osteoclast differentiation and is regulated by some miRNAs. Lou et al. found miR-142-5p could promote the bone marrow-derived macrophages differentiation by inhibiting the expression of PTEN [26]. Similarly, previous research has showed that miR-214 promoted osteoclastogenesis by targeting PTEN [17]. These results indicate that PTEN and its complete function play a crucial role in osteoclast differentiation. In the present study, overexpression of PTEN could attenuate the enhancing effects of proliferation and osteoclast differentiation of RAW264.7 cells caused by miR-455-3p-mimic. Furthermore, PTEN could also inhibit the regulating effect of cell cycle of RAW264.7 cells caused by miR-455-3p-mimic. In summary, it can be concluded that the inhibition of RAW264.7 cell proliferation and osteoclast differentiation by overexpressing miR-455-3p may be mediated by the inhibition of PTEN expression.

There was some limitation worth to be mentioned in our study. First, monocytes could differentiate into several types of cells including macrophages and osteoclasts. We only focused on the effects of miR-455-3p on the differentiation of RAW264.7 cell into osteoclasts. Although we identified miR-455-3p as a regulator of PTEN, we did not exclude other possible targets in addition to PTEN. In the present study, the results of TRAP staining including some TRAP weak positive cells. Hence, we need to obtain some clinical samples and analyze the relationship between the expression of miR-455-3p and the degree of osteoclast differentiation in the future.

## Conclusions

In summary, our results indicated that PTEN was a potential target of miR-455-3p. MiR-455-3p could promote the proliferation of RAW264.7 cells and the differentiation of RAW264.7 cells into osteoclasts induced by M-CSF/ RANKL. Overexpression of PTEN could effectively reverse the positive effect of miR-455-3p. In summary, the promotion of miR-455-3p in osteoclast proliferation and differentiation might be partly attributed to the targeted inhibition of PTEN. In this study, new roles and mechanism of miR-455-3p in RAW264.7 cell proliferation and osteoclast formation were proposed.

## Supplementary Information

**Additional file 1.**

## Data Availability

The datasets analyzed of target genes of miR-455-3p in the present study are available in miRTarBase (https://mirtarbase.cuhk.edu.cn/) and miRDB (http://mirdb.org/). The binding site of miR-455-3p in 3′-untranslated regions of PTEN was predicted using TargetScan (http://www.targetscan.org/vert_72/). Other experimental data will be available upon request to the corresponding author.

## Abbreviations

qRT-PCR: Quantitative real-time polymerase chain reaction
TRAP: Tartrate resistant acid phosphatase
ATCC: American Type Culture Collection
PI: Propyl iodide
e: Over-expression
