# Supplementary material for: MicroRNA-455-3p regulates proliferation and osteoclast differentiation of RAW264.7 cells by targeting PTEN
Source: BMC Musculoskelet Disord. 2022 Apr 9;23:340. doi: 10.1186/s12891-022-05266-0 (PMC8994399; doi:10.1186/s12891-022-05266-0)
Supplement: Supplementary file 1 — Additional file 1. [file 12891_2022_5266_MOESM1_ESM.docx]

Additional file 1: Supplement Figure 1 The direct targeted relationship between miR-455-3p and PTEN was assessed by TargetScan.


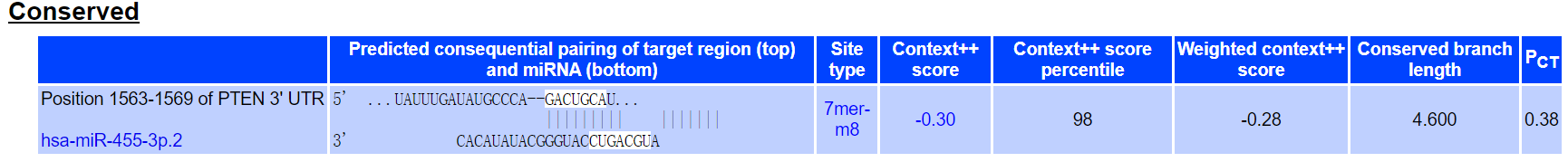


Additional file 2: Supplement Table 1 The original data of miR-455-3p target genes predicted by miRTarBase and miRDB.

| **miRTarBase ID** | **miRNA** | **Species (miRNA)** | **Target Gene** | **Target Gene (Entrez Gene ID)** | **Species (Target Gene)** | **Experiments** | **Support Type** |
| --- | --- | --- | --- | --- | --- | --- | --- |
| MIRT037803 | hsa-miR-455-3p | Homo sapiens | ND1 | 4535 | Homo sapiens | CLASH | Functional MTI (Weak) |
| MIRT037804 | hsa-miR-455-3p | Homo sapiens | STX16 | 8675 | Homo sapiens | CLASH | Functional MTI (Weak) |
| MIRT037805 | hsa-miR-455-3p | Homo sapiens | TOR1AIP2 | 163590 | Homo sapiens | CLASH | Functional MTI (Weak) |
| MIRT037806 | hsa-miR-455-3p | Homo sapiens | ND4 | 4538 | Homo sapiens | CLASH | Functional MTI (Weak) |
| MIRT037807 | hsa-miR-455-3p | Homo sapiens | EVC | 2121 | Homo sapiens | CLASH | Functional MTI (Weak) |
| MIRT037808 | hsa-miR-455-3p | Homo sapiens | COL3A1 | 1281 | Homo sapiens | CLASH | Functional MTI (Weak) |
| MIRT037809 | hsa-miR-455-3p | Homo sapiens | TMEM115 | 11070 | Homo sapiens | CLASH | Functional MTI (Weak) |
| MIRT037810 | hsa-miR-455-3p | Homo sapiens | SON | 6651 | Homo sapiens | CLASH | Functional MTI (Weak) |
| MIRT037811 | hsa-miR-455-3p | Homo sapiens | AGO1 | 26523 | Homo sapiens | CLASH | Functional MTI (Weak) |
| MIRT037812 | hsa-miR-455-3p | Homo sapiens | LYRM1 | 57149 | Homo sapiens | CLASH | Functional MTI (Weak) |
| MIRT037813 | hsa-miR-455-3p | Homo sapiens | FDX1 | 2230 | Homo sapiens | CLASH | Functional MTI (Weak) |
| MIRT037814 | hsa-miR-455-3p | Homo sapiens | TNRC18 | 84629 | Homo sapiens | CLASH | Functional MTI (Weak) |
| MIRT037815 | hsa-miR-455-3p | Homo sapiens | SRM | 6723 | Homo sapiens | CLASH | Functional MTI (Weak) |
| MIRT037816 | hsa-miR-455-3p | Homo sapiens | UTP4 | 84916 | Homo sapiens | CLASH | Functional MTI (Weak) |
| MIRT037817 | hsa-miR-455-3p | Homo sapiens | CLSTN1 | 22883 | Homo sapiens | CLASH | Functional MTI (Weak) |
| MIRT037818 | hsa-miR-455-3p | Homo sapiens | PFKP | 5214 | Homo sapiens | CLASH | Functional MTI (Weak) |
| MIRT037819 | hsa-miR-455-3p | Homo sapiens | HIST1H2BD | 3017 | Homo sapiens | CLASH | Functional MTI (Weak) |
| MIRT037820 | hsa-miR-455-3p | Homo sapiens | PWP2 | 5822 | Homo sapiens | CLASH | Functional MTI (Weak) |
| MIRT037821 | hsa-miR-455-3p | Homo sapiens | PTPRF | 5792 | Homo sapiens | CLASH | Functional MTI (Weak) |
| MIRT037821 | hsa-miR-455-3p | Homo sapiens | XPR1 | 5792 | Homo sapiens | PAR-CLIP | Functional MTI (Weak) |
| MIRT037821 | hsa-miR-455-3p | Homo sapiens | MAPRE2 | 5792 | Homo sapiens | PAR-CLIP | Functional MTI (Weak) |
| MIRT037821 | hsa-miR-455-3p | Homo sapiens | HSDL1 | 5792 | Homo sapiens | HITS-CLIP | Functional MTI (Weak) |
| MIRT037821 | hsa-miR-455-3p | Homo sapiens | TSC22D4 | 5792 | Homo sapiens | PAR-CLIP | Functional MTI (Weak) |
| MIRT037822 | hsa-miR-455-3p | Homo sapiens | LZIC | 9213 | Homo sapiens | CLASH | Functional MTI (Weak) |
| MIRT037823 | hsa-miR-455-3p | Homo sapiens | MTFR1 | 10982 | Homo sapiens | CLASH | Functional MTI (Weak) |
| MIRT037824 | hsa-miR-455-3p | Homo sapiens | BRD4 | 83693 | Homo sapiens | CLASH | Functional MTI (Weak) |
| MIRT037825 | hsa-miR-455-3p | Homo sapiens | GBA2 | 81628 | Homo sapiens | CLASH | Functional MTI (Weak) |
| MIRT037826 | hsa-miR-455-3p | Homo sapiens | SHCBP1 | 84328 | Homo sapiens | CLASH | Functional MTI (Weak) |
| MIRT037827 | hsa-miR-455-3p | Homo sapiens | SRRM2 | 9650 | Homo sapiens | CLASH | Functional MTI (Weak) |
| MIRT037828 | hsa-miR-455-3p | Homo sapiens | MGRN1 | 23476 | Homo sapiens | CLASH | Functional MTI (Weak) |
| MIRT037829 | hsa-miR-455-3p | Homo sapiens | EIF2B2 | 57704 | Homo sapiens | CLASH | Functional MTI (Weak) |
| MIRT037830 | hsa-miR-455-3p | Homo sapiens | ACTB | 79801 | Homo sapiens | CLASH | Functional MTI (Weak) |
| MIRT037831 | hsa-miR-455-3p | Homo sapiens | SYNGR2 | 23524 | Homo sapiens | CLASH | Functional MTI (Weak) |
| MIRT037832 | hsa-miR-455-3p | Homo sapiens | CUX1 | 23295 | Homo sapiens | CLASH | Functional MTI (Weak) |
| MIRT037833 | hsa-miR-455-3p | Homo sapiens | RRP1B | 8892 | Homo sapiens | CLASH | Functional MTI (Weak) |
| MIRT037834 | hsa-miR-455-3p | Homo sapiens | DYNC1H1 | 60 | Homo sapiens | CLASH | Functional MTI (Weak) |
| MIRT037835 | hsa-miR-455-3p | Homo sapiens | EIF4G1 | 9144 | Homo sapiens | CLASH | Functional MTI (Weak) |
| MIRT037836 | hsa-miR-455-3p | Homo sapiens | RRP7A | 1523 | Homo sapiens | CLASH | Functional MTI (Weak) |
| MIRT037837 | hsa-miR-455-3p | Homo sapiens | SCD | 23076 | Homo sapiens | CLASH | Functional MTI (Weak) |
| MIRT037838 | hsa-miR-455-3p | Homo sapiens | MAP1B | 1778 | Homo sapiens | CLASH | Functional MTI (Weak) |
| MIRT037839 | hsa-miR-455-3p | Homo sapiens | RPS27 | 1981 | Homo sapiens | CLASH | Functional MTI (Weak) |
| MIRT037840 | hsa-miR-455-3p | Homo sapiens | GALM | 27341 | Homo sapiens | CLASH | Functional MTI (Weak) |
| MIRT037840 | hsa-miR-455-3p | Homo sapiens | CAD | 27341 | Homo sapiens | PAR-CLIP | Functional MTI (Weak) |
| MIRT037841 | hsa-miR-455-3p | Homo sapiens | AP1B1 | 6319 | Homo sapiens | CLASH | Functional MTI (Weak) |
| MIRT037842 | hsa-miR-455-3p | Homo sapiens | NISCH | 4131 | Homo sapiens | CLASH | Functional MTI (Weak) |
| MIRT037843 | hsa-miR-455-3p | Homo sapiens | NT5DC2 | 6232 | Homo sapiens | CLASH | Functional MTI (Weak) |
| MIRT037844 | hsa-miR-455-3p | Homo sapiens | USP10 | 130589 | Homo sapiens | CLASH | Functional MTI (Weak) |
| MIRT037845 | hsa-miR-455-3p | Homo sapiens | AGL | 790 | Homo sapiens | CLASH | Functional MTI (Weak) |
| MIRT037845 | hsa-miR-455-3p | Homo sapiens | ADGRL2 | 790 | Homo sapiens | PAR-CLIP | Functional MTI (Weak) |
| MIRT037846 | hsa-miR-455-3p | Homo sapiens | TFE3 | 162 | Homo sapiens | CLASH | Functional MTI (Weak) |
| MIRT037847 | hsa-miR-455-3p | Homo sapiens | HIST1H1E | 11188 | Homo sapiens | CLASH | Functional MTI (Weak) |
| MIRT037848 | hsa-miR-455-3p | Homo sapiens | LRP11 | 64943 | Homo sapiens | CLASH | Functional MTI (Weak) |
| MIRT037849 | hsa-miR-455-3p | Homo sapiens | INTS4 | 9100 | Homo sapiens | CLASH | Functional MTI (Weak) |
| MIRT037850 | hsa-miR-455-3p | Homo sapiens | BTF3 | 178 | Homo sapiens | CLASH | Functional MTI (Weak) |
| MIRT037851 | hsa-miR-455-3p | Homo sapiens | UTRN | 23266 | Homo sapiens | CLASH | Functional MTI (Weak) |
| MIRT037852 | hsa-miR-455-3p | Homo sapiens | PRPF40A | 7030 | Homo sapiens | CLASH | Functional MTI (Weak) |
| MIRT037853 | hsa-miR-455-3p | Homo sapiens | BPTF | 3008 | Homo sapiens | CLASH | Functional MTI (Weak) |
| MIRT037854 | hsa-miR-455-3p | Homo sapiens | HSP90AB1 | 84918 | Homo sapiens | CLASH | Functional MTI (Weak) |
| MIRT037855 | hsa-miR-455-3p | Homo sapiens | BMPR1A | 92105 | Homo sapiens | CLASH | Functional MTI (Weak) |
| MIRT037856 | hsa-miR-455-3p | Homo sapiens | MAEA | 689 | Homo sapiens | CLASH | Functional MTI (Weak) |
| MIRT037857 | hsa-miR-455-3p | Homo sapiens | VPS4A | 7402 | Homo sapiens | CLASH | Functional MTI (Weak) |
| MIRT037858 | hsa-miR-455-3p | Homo sapiens | BCL2L12 | 55660 | Homo sapiens | CLASH | Functional MTI (Weak) |
| MIRT037859 | hsa-miR-455-3p | Homo sapiens | ATP6AP1 | 2186 | Homo sapiens | CLASH | Functional MTI (Weak) |
| MIRT037860 | hsa-miR-455-3p | Homo sapiens | RPL8 | 3326 | Homo sapiens | CLASH | Functional MTI (Weak) |
| MIRT037861 | hsa-miR-455-3p | Homo sapiens | RPS15A | 657 | Homo sapiens | CLASH | Functional MTI (Weak) |
| MIRT037861 | hsa-miR-455-3p | Homo sapiens | RPS5 | 657 | Homo sapiens | PAR-CLIP | Functional MTI (Weak) |
| MIRT037862 | hsa-miR-455-3p | Homo sapiens | CRIM1 | 10296 | Homo sapiens | CLASH | Functional MTI (Weak) |
| MIRT037863 | hsa-miR-455-3p | Homo sapiens | SIN3A | 27183 | Homo sapiens | CLASH | Functional MTI (Weak) |
| MIRT037864 | hsa-miR-455-3p | Homo sapiens | DAZAP2 | 83596 | Homo sapiens | CLASH | Functional MTI (Weak) |
| MIRT037864 | hsa-miR-455-3p | Homo sapiens | HMGN2 | 83596 | Homo sapiens | PAR-CLIP | Functional MTI (Weak) |
| MIRT037865 | hsa-miR-455-3p | Homo sapiens | N4BP2L2 | 537 | Homo sapiens | CLASH | Functional MTI (Weak) |
| MIRT037866 | hsa-miR-455-3p | Homo sapiens | BAIAP2 | 6132 | Homo sapiens | CLASH | Functional MTI (Weak) |
| MIRT037867 | hsa-miR-455-3p | Homo sapiens | PLEKHM2 | 6210 | Homo sapiens | CLASH | Functional MTI (Weak) |
| MIRT037868 | hsa-miR-455-3p | Homo sapiens | CISD2 | 6193 | Homo sapiens | CLASH | Functional MTI (Weak) |
| MIRT037869 | hsa-miR-455-3p | Homo sapiens | KPNB1 | 51232 | Homo sapiens | CLASH | Functional MTI (Weak) |
| MIRT037870 | hsa-miR-455-3p | Homo sapiens | ERI3 | 25942 | Homo sapiens | CLASH | Functional MTI (Weak) |
| MIRT037871 | hsa-miR-455-3p | Homo sapiens | EIF3D | 9802 | Homo sapiens | CLASH | Functional MTI (Weak) |
| MIRT037872 | hsa-miR-455-3p | Homo sapiens | POLR2B | 3151 | Homo sapiens | CLASH | Functional MTI (Weak) |
| MIRT037873 | hsa-miR-455-3p | Homo sapiens | YRDC | 10443 | Homo sapiens | CLASH | Functional MTI (Weak) |
| MIRT037874 | hsa-miR-455-3p | Homo sapiens | RAD23A | 10458 | Homo sapiens | CLASH | Functional MTI (Weak) |
| MIRT037875 | hsa-miR-455-3p | Homo sapiens | SAT1 | 23207 | Homo sapiens | CLASH | Functional MTI (Weak) |
| MIRT037876 | hsa-miR-455-3p | Homo sapiens | RANBP3 | 493856 | Homo sapiens | CLASH | Functional MTI (Weak) |
| MIRT037877 | hsa-miR-455-3p | Homo sapiens | PGM3 | 3837 | Homo sapiens | CLASH | Functional MTI (Weak) |
| MIRT037878 | hsa-miR-455-3p | Homo sapiens | WRAP53 | 79033 | Homo sapiens | CLASH | Functional MTI (Weak) |
| MIRT037879 | hsa-miR-455-3p | Homo sapiens | EEF1A1 | 8664 | Homo sapiens | CLASH | Functional MTI (Weak) |
| MIRT037880 | hsa-miR-455-3p | Homo sapiens | PGRMC1 | 5431 | Homo sapiens | CLASH | Functional MTI (Weak) |
| MIRT037881 | hsa-miR-455-3p | Homo sapiens | HIST2H3A | 79693 | Homo sapiens | CLASH | Functional MTI (Weak) |
| MIRT037882 | hsa-miR-455-3p | Homo sapiens | HNRNPC | 5886 | Homo sapiens | CLASH | Functional MTI (Weak) |
| MIRT037883 | hsa-miR-455-3p | Homo sapiens | YWHAZ | 6303 | Homo sapiens | CLASH | Functional MTI (Weak) |
| MIRT037884 | hsa-miR-455-3p | Homo sapiens | DYRK1B | 8498 | Homo sapiens | CLASH | Functional MTI (Weak) |
| MIRT037885 | hsa-miR-455-3p | Homo sapiens | U2AF2 | 5238 | Homo sapiens | CLASH | Functional MTI (Weak) |
| MIRT037886 | hsa-miR-455-3p | Homo sapiens | WBP2 | 55135 | Homo sapiens | CLASH | Functional MTI (Weak) |
| MIRT037887 | hsa-miR-455-3p | Homo sapiens | RNF216 | 1915 | Homo sapiens | CLASH | Functional MTI (Weak) |
| MIRT037887 | hsa-miR-455-3p | Homo sapiens | HERC1 | 1915 | Homo sapiens | PAR-CLIP | Functional MTI (Weak) |
| MIRT037888 | hsa-miR-455-3p | Homo sapiens | CDC45 | 10857 | Homo sapiens | CLASH | Functional MTI (Weak) |
| MIRT037889 | hsa-miR-455-3p | Homo sapiens | HYOU1 | 333932 | Homo sapiens | CLASH | Functional MTI (Weak) |
| MIRT037890 | hsa-miR-455-3p | Homo sapiens | PTEN | 5728 | Homo sapiens | CLASH | Functional MTI (Weak) |
| MIRT037891 | hsa-miR-455-3p | Homo sapiens | PPIA | 3183 | Homo sapiens | CLASH | Functional MTI (Weak) |
| MIRT037891 | hsa-miR-455-3p | Homo sapiens | RPS2 | 7534 | Homo sapiens | CLASH | Functional MTI (Weak) |
| MIRT037892 | hsa-miR-455-3p | Homo sapiens | GIGYF2 | 7534 | Homo sapiens | PAR-CLIP | Functional MTI (Weak) |
| MIRT037893 | hsa-miR-455-3p | Homo sapiens | SLC4A2 | 9149 | Homo sapiens | CLASH | Functional MTI (Weak) |
| MIRT037894 | hsa-miR-455-3p | Homo sapiens | LIMD1 | 11338 | Homo sapiens | CLASH | Functional MTI (Weak) |
| MIRT037895 | hsa-miR-455-3p | Homo sapiens | ELOVL1 | 23558 | Homo sapiens | CLASH | Functional MTI (Weak) |
| MIRT037896 | hsa-miR-455-3p | Homo sapiens | EPHA7 | 54476 | Homo sapiens | CLASH | Functional MTI (Weak) |
| MIRT037897 | hsa-miR-455-3p | Homo sapiens | NDUFS7 | 8925 | Homo sapiens | CLASH | Functional MTI (Weak) |
| MIRT037898 | hsa-miR-455-3p | Homo sapiens | NPTN | 8318 | Homo sapiens | CLASH | Functional MTI (Weak) |
| MIRT037899 | hsa-miR-455-3p | Homo sapiens | DDX55 | 10525 | Homo sapiens | CLASH | Functional MTI (Weak) |
| MIRT037900 | hsa-miR-455-3p | Homo sapiens | USP5 | 5478 | Homo sapiens | CLASH | Functional MTI (Weak) |
| MIRT037901 | hsa-miR-455-3p | Homo sapiens | CDKN2A | 6187 | Homo sapiens | CLASH | Functional MTI (Weak) |
| MIRT037902 | hsa-miR-455-3p | Homo sapiens | RHOC | 26058 | Homo sapiens | CLASH | Functional MTI (Weak) |
| MIRT037903 | hsa-miR-455-3p | Homo sapiens | INTS10 | 6522 | Homo sapiens | CLASH | Functional MTI (Weak) |

Additional file 3: The original data of qRT-PCR, MTT, double fluorescence report and TRAP.

| Figure1A | | | | | | | | | | | | |
| --- | --- | --- | --- | --- | --- | --- | --- | --- | --- | --- | --- | --- |
| **Control** | | **NC-mimic** | | **miR-455-3p-mimic** | |  | **Control** | | **NC-inhibitor** | | **miR-455-3p-inhibitor** | |
| miR-455-3p | U6 | miR-455-3p | U6 | miR-455-3p | U6 |  | miR-455-3p | U6 | miR-455-3p | U6 | miR-455-3p | U6 |
| 28.18 | 18.3 | 28.44 | 18.78 | 25.42 | 18.26 |  | 28.57 | 18.73 | 28.56 | 18.68 | 29.95 | 18.9 |
| 28.47 | 18.85 | 28.31 | 18.89 | 25.75 | 18.83 |  | 27.59 | 18.02 | 27.9 | 18.3 | 29.69 | 18.88 |
| 28.17 | 18.04 | 28.81 | 18.86 | 26.02 | 18.59 |  | 28.23 | 18.11 | 27.96 | 17.82 | 29.74 | 18.42 |
| Figure1B | | | | | | | | | | | | |
|  | 0d | 1d | 2d | 3d | 4d |  |  | 0d | 1d | 2d | 3d | 4d |
| OD （Control） | 0.311 | 0.442 | 0.733 | 1.043 | 0.978 |  | OD （Control） | 0.289 | 0.337 | 0.638 | 0.919 | 0.927 |
|  | 0.381 | 0.482 | 0.66 | 0.704 | 1.045 |  |  | 0.308 | 0.398 | 0.746 | 0.916 | 0.955 |
|  | 0.364 | 0.499 | 0.666 | 0.823 | 0.965 |  |  | 0.316 | 0.425 | 0.705 | 0.992 | 1.092 |
| OD （NC-mimic） | 0.324 | 0.357 | 0.651 | 0.789 | 0.948 |  | OD （NC-inhibitor） | 0.289 | 0.456 | 0.729 | 0.772 | 0.993 |
|  | 0.365 | 0.432 | 0.521 | 0.759 | 0.893 |  |  | 0.311 | 0.407 | 0.621 | 0.86 | 0.914 |
|  | 0.29 | 0.362 | 0.599 | 0.801 | 1.038 |  |  | 0.292 | 0.511 | 0.651 | 0.794 | 0.961 |
| OD （miR-455-3p-mimic） | 0.383 | 0.558 | 0.837 | 0.854 | 1.27 |  | OD （miR-455-3p-inhibitor） | 0.3 | 0.326 | 0.487 | 0.633 | 0.699 |
|  | 0.422 | 0.493 | 0.784 | 0.927 | 1.379 |  |  | 0.3 | 0.464 | 0.411 | 0.627 | 0.779 |
|  | 0.363 | 0.44 | 0.816 | 0.97 | 1.358 |  |  | 0.318 | 0.474 | 0.574 | 0.68 | 0.678 |
|  |  |  |  |  |  |  |  |  |  |  |  |  |
| Figure2B | | | | | | |  | Figure2C | | | | |
| PTEN | **Control** | | **miR-455-3p-mimic** | | **miR-455-3p-inhibitor** | |  |  |  |  |  |  |
|  | 28.06 | 18.26 | 30.18 | 18.89 | 28.2 | 18.86 |  |  | WT-PTEN |  | MUT-PTEN |  |
|  | 27.72 | 18.13 | 29.87 | 18.86 | 27.92 | 18.8 |  |  | NC-mimic | miR-455-3p mimic | NC-mimic | miR-455-3p mimic |
|  | 28.88 | 18.74 | 29.6 | 18.01 | 27.97 | 18.27 |  | fireflyluciferase | 42679.46 | 22620.11 | 40545.49 | 43533.05 |
| BCL2L12 | **Control** | | **miR-455-3p-mimic** | | **miR-455-3p-inhibitor** | |  |  | 45752.38 | 24668.73 | 44642.72 | 48313.15 |
|  | 27.85 | 18.02 | 28.41 | 18.68 | 27.81 | 18.11 |  |  | 39606.54 | 20571.5 | 36448.26 | 38752.95 |
|  | 27.51 | 17.89 | 27.66 | 18.19 | 27.63 | 18.16 |  | Renilla luciferase | 534533.43 | 533494.8 | 537526.6 | 536875.33 |
|  | 28.29 | 18.14 | 28.46 | 18.43 | 28.54 | 18.55 |  |  | 529669.4 | 538426.9 | 528815.11 | 529516.72 |
| GIGYF2 | **Control** | | **miR-455-3p-mimic** | | **miR-455-3p-inhibitor** | |  |  | 529581.3 | 537742.9 | 534523.78 | 535632.85 |
|  | 28.21 | 18.37 | 27.95 | 18.15 | 28.7 | 18.8 |  |  |  |  |  |  |
|  | 27.66 | 18.07 | 27.64 | 18.05 | 27.77 | 18.1 |  |  |  |  |  |  |
|  | 28.24 | 18.13 | 28.82 | 18.69 | 28.98 | 18.77 |  |  |  |  |  |  |
|  |  |  |  |  |  |  |  |  |  |  |  |  |
| Figure 3A | | | | | | | |  |  |  |  |  |
| **Control** | | **miR-455-3p-mimic** | | oe-PTNE | | **miR-455-3p-mimic+oe-PTEN** | |  |  |  |  |  |
| 28.4 | 18.56 | 25.91 | 18.12 | 28.56 | 18.65 | 26.26 | 18.42 |  |  |  |  |  |
| 28.26 | 18.67 | 25.84 | 18.31 | 27.6 | 17.92 | 26.16 | 18.55 |  |  |  |  |  |
| 28.31 | 18.19 | 26.27 | 18.2 | 28.96 | 18.72 | 26.76 | 18.61 |  |  |  |  |  |
| Figure 3C | | | | | | | |  |  |  |  |  |
| **Control** |  | **miR-455-3p-mimic** |  | oe-PTNE |  | **miR-455-3p-mimic+oe-PTEN** |  |  |  |  |  |  |
| 28.03 | 18.19 | 29.61 | 17.98 | 27.5 | 18.66 | 29.07 | 18.38 |  |  |  |  |  |
| 27.97 | 18.37 | 30.05 | 18.68 | 27.02 | 18.39 | 28.64 | 18.17 |  |  |  |  |  |
| 28.56 | 18.41 | 30.55 | 18.64 | 27.95 | 18.81 | 29.57 | 18.58 |  |  |  |  |  |
| Figure 3D | | | | | | | |  |  |  |  |  |
|  | 0d | 1d | 2d | 3d | 4d |  |  |  |  |  |  |  |
| OD （Control） | 0.398 | 0.359 | 0.504 | 0.826 | 0.913 |  |  |  |  |  |  |  |
|  | 0.256 | 0.275 | 0.611 | 0.703 | 0.865 |  |  |  |  |  |  |  |
|  | 0.324 | 0.396 | 0.532 | 0.852 | 0.985 |  |  |  |  |  |  |  |
| OD （miR-455-3p-mimic） | 0.392 | 0.527 | 0.676 | 1.055 | 1.261 |  |  |  |  |  |  |  |
|  | 0.395 | 0.562 | 0.817 | 1.11 | 1.499 |  |  |  |  |  |  |  |
|  | 0.356 | 0.548 | 0.831 | 1.048 | 1.32 |  |  |  |  |  |  |  |
| OD （oe-PTEN） | 0.392 | 0.361 | 0.448 | 0.605 | 0.735 |  |  |  |  |  |  |  |
|  | 0.376 | 0.421 | 0.389 | 0.576 | 0.785 |  |  |  |  |  |  |  |
|  | 0.473 | 0.468 | 0.474 | 0.53 | 0.657 |  |  |  |  |  |  |  |
| OD （miR-455-3p-mimic+oe-PTEN） | 0.387 | 0.486 | 0.584 | 0.768 | 0.906 |  |  |  |  |  |  |  |
|  | 0.363 | 0.511 | 0.663 | 0.842 | 1.154 |  |  |  |  |  |  |  |
|  | 0.437 | 0.464 | 0.577 | 0.945 | 1.087 |  |  |  |  |  |  |  |
| Figure 4A |  |  |  |  |  |  |  |  |  |  |  |  |
|  |  |  |  |  |  |  |  |  |  |  |  |  |
| control | RANKL/M-CSF | RANKL/M-CSF+miR-455-3p-mimic | RANKL/M-CSF+miR-455-3p-mimic+oe-PTEN | | | |  |  |  |  |  |  |
| 16 | 28 | 65 | 36 |  |  |  |  |  |  |  |  |  |
| 11 | 26 | 65 | 39 |  |  |  |  |  |  |  |  |  |
| 8 | 35 | 61 | 35 |  |  |  |  |  |  |  |  |  |

Additional file 4: Original Image of Western blot

TRAP of Figure 1E and Figure 4B





**oe-PTEN**

**miR-455-3p-mimic**

**RANKL/M-CSF**

**+**

**+**

**+**

**+**

**+**

**-**

**+**

**-**

**-**

**-**

**-**

**-**

**miR-455-3p-inhibitor**

**miR-455-3p-mimic**

**RANKL/M-CSF**

**-**

**-**

**+**

**+**

**-**

**-**

**+**

**+**

**+**

**-**

**-**

**-**

**TRAP**

CTSK of Figure 1E and Figure4B





**+**

**-**

**-**

**+**

**+**

**-**

**+**

**+**

**+**

**-**

**-**

**-**

**+**

**-**

**-**

**+**

**+**

**-**

**+**

**+**

**-**

**-**

**-**

**-**

**miR-455-3p-inhibitor**

**miR-455-3p-mimic**

**RANKL/M-CSF**

**RANKL/M-CSF**

**miR-455-3p-mimic**

**oe-PTEN**

**CTSK**

Cyclin D1 of Figure 3B





**Cyclin D1**

**miR-455-3p-mimic**

**oe-PTEN**

**-**

**-**

**+**

**-**

**+**

**+**

**-**

**+**

GAPDH of Figure 3B and Figure 2D





**GAPDH**

**miR-455-3p-mimic**

**oe-PTEN**

**-**

**-**

**+**

**-**

**+**

**+**

**-**

**+**

**miR-455-3p-mimic**

**miR-455-3p-inhibitor**

**-**

**-**

**+**

**+**

**-**

**-**

GAPDH of Figure 1E and Figure 4B





**RANKL/M-CSF**

**miR-455-3p-mimic**

**miR-455-3p-inhibitor**

**RANKL/M-CSF**

**miR-455-3p-mimic**

**oe-PTEN**

**+**

**-**

**-**

**+**

**+**

**-**

**+**

**+**

**+**

**-**

**-**

**-**

**+**

**-**

**-**

**+**

**+**

**-**

**+**

**+**

**-**

**-**

**-**

**-**

**GAPDH**

NFATc1 of Figure 1E and Figure 4B





**RANKL/M-CSF**

**miR-455-3p-mimic**

**miR-455-3p-inhibitor**

**RANKL/M-CSF**

**miR-455-3p-mimic**

**oe-PTEN**

**+**

**-**

**-**

**+**

**+**

**-**

**+**

**+**

**+**

**-**

**-**

**-**

**+**

**-**

**-**

**+**

**+**

**-**

**+**

**+**

**-**

**-**

**-**

**-**

**NFATc1**

p-AKT of Figure 3B





**P-AKT**

**-**

**+**

**+**

**+**

**+**

**-**

**-**

**-**

**oe-PTEN**

**miR-455-3p-mimic**

PTEN of Figure 4B and Figure 2D





**RANKL/M-CSF**

**miR-455-3p-mimic**

**oe-PTEN**

**+**

**-**

**-**

**+**

**+**

**-**

**+**

**+**

**+**

**-**

**-**

**-**

**miR-455-3p-mimic**

**miR-455-3p-inhibitor**

**-**

- **-**

**+**

**+**

**-**

**PTEN**

PTEN of Figure 3B





**miR-455-3p-mimic**

**oe-PTEN**

**-**

**-**

**+**

**-**

**+**

**+**

**-**

**+**

**PTEN**
